# Stressor interactions affect myxozoan abundance in a 42-year dataset from the Pearl River, Louisiana, USA

**DOI:** 10.1017/S0031182026101917

**Published:** 2026-04

**Authors:** Dakeishla M. Díaz-Morales, Stephen D. Atkinson, Desmond Boyd, Gabriella Commisso, Shyanne R. Christner, Imani Jones, Katie L. Leslie, Jolee Thirtyacre, Connor J. Whalen, Armand Kuris, Justin Mann, Henry L. Bart, Chelsea L. Wood

**Affiliations:** 1 Department of Biological Sciences, Depaul University, Chicago, IL, USA; 2 School of Aquatic and Fishery Sciences, University of Washington, Seattle, WA, USA; 3 Department of Microbiology, Oregon State University, Corvallis, OR, USA; 4 School of the Earth, Ocean and Environment, University of South Carolina, Columbia, SC, USA; 5 Department of Biology, Valdosta State University, Valdosta, GA, USA; 6 College of Agriculture and Environmental Sciences, Tuskegee University, AL, USA; 7 Department of Ecology, Evolution, and Marine Biology, University of California Santa Barbara, CA, USA; 8 Biodiversity Research Institute, Tulane University, New Orleans, LA, USA; 9 Department of Ecology and Evolutionary Biology, Tulane University, New Orleans, LA, USA

**Keywords:** fish, freshwater, historical ecology, warming

## Abstract

Environmental change can impact host–parasite interactions, but the effects of multiple stressors on parasites are rarely measured. Considering stressor interactions may allow parasitologists to evaluate how parasite burdens change in nature, where stressors rarely occur in isolation. This study aimed to understand how combined stressors such as warming, nutrients and pollution (i.e. metal concentrations) influence myxozoan prevalence and abundance in the Pearl River, Louisiana, USA. Fish were seined between 1963 and 2005 upstream and downstream of a pulp-mill outfall and were then preserved and accessioned into the Royal D. Suttkus Fish Collection of the Tulane University Biodiversity Research Institute. In 2024, we dissected 1188 fish individuals across 7 host species, and we identified myxozoans in 6 species. Six myxozoan genera were detected, including *Chloromyxum, Henneguya, Myxidium, Myxobolus, Thelohanellus* and *Unicauda*, with some novel host–parasite combinations. The abundance of *Myxobolus* infecting *Carpiodes velifer* gills declined by 86% over the study period, while the abundance of *Myxobolus* infecting *Pimephales vigilax* gills was significantly lower downstream of the pulp mill outfall. Among the drivers analyzed, temperature had a significant negative effect on this parasite’s abundance, metal concentrations had a positive effect, and these 2 drivers interacted. Our results highlight the differential susceptibility of wild fishes to myxozoan infections and the usefulness of museum collections for understanding historical change in myxozoan burdens in fish. Since stressor-driven changes in myxozoan abundance do not follow a single pattern across species, we expect a shift in freshwater myxozoan communities with progressing climate change and pollution.

## Graphical abstract

**Figure:**
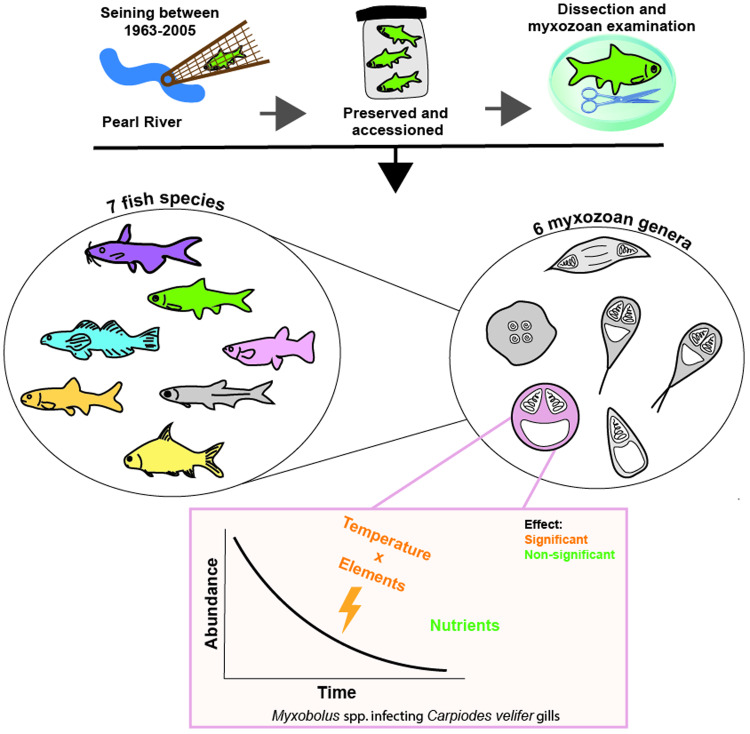

## Introduction

Freshwater ecosystems experience many anthropogenic impacts, or stressors, simultaneously (Ormerod et al., 2010; Lemm et al., 2021). Published studies often evaluate multiple stressor impacts on free-living aquatic organisms (Birk et al., 2020), but parasites are considered in only 2.3% of these studies (Clarivate, 2024), even though parasites represent a large proportion of biodiversity (Goater et al., 2014; Poulin, 2014) and play essential roles in ecosystems (Lafferty et al., 2006, 2008; Kuris et al., 2008; Dunne et al., 2013; Moore et al., 2024). Parasites contribute to standing stock biomass (Kuris et al., 2008), control host populations that would otherwise increase (Dunn and Smith, 2001), modulate food web complexity, robustness and energy flow (Lafferty et al., 2008; Dunne et al., 2013; Moore et al., 2024), and change ecotoxicological dynamics (Nachev et al., 2013; Sures et al., 2017). If multiple stressors act to facilitate parasites, this is something we might wish to know so that we can mitigate the impacts of enhanced parasite transmission; if multiple stressors reduce parasite transmission, it would behoove us to understand the implications of parasite biodiversity loss for ecosystem function. Therefore, understanding how multiple stressors affect parasites is essential when assessing aquatic ecosystem health and integrity.

Myxozoans are of particular interest in the context of multiple stressors. They are ubiquitous and diverse parasites belonging to Phylum Cnidaria and subdivided into 2 classes: Myxosporea and Malacosporea (Atkinson et al., 2018). Myxozoans have complex life cycles and use fishes as intermediate hosts and annelids (Myxosporea) or bryozoans (Malacosporea) as definitive hosts, although some species can use amphibians, elasmobranchs, birds, monogeneans or mammals in lieu of fish (Alama-Bermejo and Holzer, 2021; Kmentová et al., 2025). In their vertebrate hosts, myxozoans can be found as floating spores in fluids (e.g. bile, urine), in the integument, as pseudocysts embedded in tissue (e.g. skin, gills, muscles), in the body cavity, or systemically (Lom and Dyková, 2006). The parasite proliferates asexually and produces spores that are released into the water column through bursting pseudocysts, by release of body fluids (Hedrick et al., 2004), or post-mortem through host tissue decomposition. After the spores are released, they infect an invertebrate (i.e. bryozoans or annelids), in which they produce the next phase of spores (Lom and Dyková, 2006). These spores are released into the water column, where they come into contact with fish. Myxozoans can be problematic for fisheries (see Holzer et al. (2021)). For instance, the myxozoans *Kudoa* spp., *Myxobolus cerebralis* and *Tetracapsuloides bryosalmonae* can cause myoliquefaction (‘jelly flesh’), whirling disease and proliferative kidney disease, respectively, having major consequences in aquaculture and for wild fish. Young-of-year fish are particularly sensitive and susceptible to infections (Holzer et al., 2021). While the ecological roles of myxozoans still require elucidation, these infections and resulting pathologies occur in the wild (Giulietti et al., 2022; Banu and Rathinam, 2023), influencing wild fish populations (Jones et al., 2015). Moreover, myxozoans constitute at least 19% of all described cnidarian species (Atkinson et al., 2018), and thus contribute many species to global biodiversity counts. Given their economic and ecological importance, understanding the influence of environmental change on the transmission of myxozoans is essential.

Temperature is one of the most important factors regulating biological activity, including that of parasites and their hosts (Molnár et al., 2017). Temperature effects on host–parasite interactions can be host- or parasite-mediated effects. Host-mediated effects include an increase in protective mechanisms such as heat shock proteins (Somero, 2002; Dammark et al., 2018), and accelerated immunological reactions under benign temperatures, which might protect the host from infections (Matozzo et al., 2011; Labaude et al., 2017). However, at suboptimal temperatures, hosts might experience thermal stress manifested as increased compensatory feeding, which can hamper defence against parasites by increasing the contact between hosts and parasites, or through impaired defensive metabolic responses at sub-lethal temperatures that compromise host immunocompetence (Sokolova et al., 2012). Temperatures lethal to the host can result in 2 different scenarios. For some parasite species, the host’s death can be detrimental for the parasite, particularly for those with complex life cycles, where a missing host will introduce a bottleneck into the parasite’s life cycle. For other parasite species, the host’s death can be fortuitous, in the sense that it allows for tissue decomposition, spore dispersal and transmission. In terms of parasite-mediated effects, optimal temperatures can accelerate the production of free-living larval stages and increase their infectivity, which promotes parasite transmission (Morley and Lewis, 2013, 2015; Molnár et al., 2017). Nevertheless, temperature can also be lethal directly to the parasite, interrupting transmission to downstream hosts (Morley, 2011; Díaz-Morales et al., 2022). As it is for other parasites, temperature is a strong determinant of myxozoan development. At lower temperatures, myxozoan infections are arrested, while increasing temperatures speed parasite replication, development and transmission (Okamura et al., 2011; Strepparava et al., 2018; Bolin et al., 2024).

Changes in water chemistry, such as increased concentrations of metals and metalloids (hereafter, ‘metals’) and nutrients, have repercussions for host–parasite interactions (Sures et al., 2017). Pollutants can amplify disease by compromising the immune system of the host and increasing its susceptibility to parasite infections (reviewed in Morley et al. (2006)) or conversely can reduce disease by killing or lowering infectivity of free-living stages (reviewed in Sures et al. (2017)). Moreover, parasites such as acanthocephalans, cestodes and nematodes can interfere with ecotoxicological dynamics by acting as pollutant sinks and decreasing the pollutant burden within the host (Bergey et al., 2002; Baruš et al., 2012; Filipović Marijić et al., 2014). While this line of research is picking up pace for several parasite groups, not much is known about the role of myxozoans in ecotoxicological dynamics. On the other hand, the impact of nutrients on myxozoan infections has been better researched (McKenzie and Townsend, 2007). Nutrients increase system productivity and enhance the reproduction of annelids and bryozoans (Aston, 1973). This can benefit myxozoans indirectly by increasing the number of hosts available to complete their life cycle (Kaeser et al., 2006; Marcogliese and Cone, 2021). A similar association was observed downstream from a sewage outflow, where higher myxozoan prevalence and annelid abundance were observed (Marcogliese and Cone, 2001; Marcogliese et al., 2009).

Stressors such as warming and pollutants can interact, resulting in synergistic or antagonistic impacts on aquatic biota. A study performed in European freshwater ecosystems showed that additive and interactive effects constitute 51% of the explained variance across rivers (Lemm et al., 2021). In addition, stressors are not static: global warming is progressing through time, and changes in water chemistry are dynamic with periods of high input of pollutants into waterways, depending on factors such as precipitation, intensity of human activities and policy. The dynamism of chemical changes and the progression of warming make it imperative to study how these stressors interact. While targeted short-term experimental studies can help to elucidate the mechanisms behind environmental change and parasite abundance relationships, such designs tend to have a few weaknesses: (1) a focus only on a subset of easily manipulable drivers, since it is logistically difficult to replicate the complex combinations of stressors that occur in nature; (2) a focus on ‘high-profile’ myxozoan species relevant to fisheries and aquaculture (e.g. *Myxobolus cerebralis* and *Ceratonova shasta*), which fails to recognize other species that could react differently to environmental change; (3) no natural, historical baseline on myxozoan abundance, which can be problematic for management and empirical studies seeking to reach conclusions of whether ecosystems contain more or fewer parasites than in the past (Wood, 2025). Long-term studies in the field allow us to address these questions while accounting for the complex interaction of abiotic variables and parasite species, thus increasing the generality of results. One approach is the use of historical museum collections (Wood and Vanhove, 2023). Museums hold millions of potential host specimens (e.g. fish) preserved in fluid together with their parasites. When collections are well curated, parasites can be recovered and identified morphologically (Wood et al., 2023a). Therefore, accessing these collections unlocks an opportunity to quantify how parasite burdens have changed over time, and, along with historical data on abiotic variables (when available), we can erect databases that allow us to elucidate potential drivers of change in myxozoan abundance (Welicky et al., 2021; Wood et al., 2023b).

The Royal D. Suttkus Fish Collection at the Tulane University Biodiversity Research Institute (TUBRI) in New Orleans, Louisiana, USA, presented us with an opportunity to understand how multiple stressors affect myxozoan prevalence and abundance in their fish hosts. Between 1963 and 2005, ichthyologist Royal D. Suttkus PhD made it one of his life projects to study how fish communities in the Pearl River were affected by anthropogenic activities such as the pulp mill industry. This river encompasses habitat critical for threatened species such as the Gulf sturgeon, *Acipenser oxyrinchus desotoi*, and supports local fisheries, recreational activities and oyster reefs. However, pulp mills were not the only industry around Pearl River; agriculture-related activities (e.g. poultry and farming) and the oil and natural gas industry were also dominant during this period (Lang, 1972). Moreover, global warming puts additional progressing pressure on surface waters.

Using specimens from the Royal D. Suttkus Fish Collection, we aimed to determine the single and combined effects of temperature (i.e. warming), and metal and nutrient concentration on myxozoan prevalence and abundance in freshwater fishes (*Carpiodes velifer, Gambusia affinis, Hybognathus nuchalis, Ictalurus punctatus, Notropis atherinoides, Percina vigil* and *Pimephales vigilax*). Specifically, we addressed: (1) whether myxozoan prevalence and abundance increased or decreased between 1963 and 2005; and (2) how pollutants from point and nonpoint sources (metals and nutrients) and temperature interacted to influence change in myxozoan prevalence and abundance in fishes over this timespan.

## Methods

### Specimen selection and collection site history

**Figure 1.**
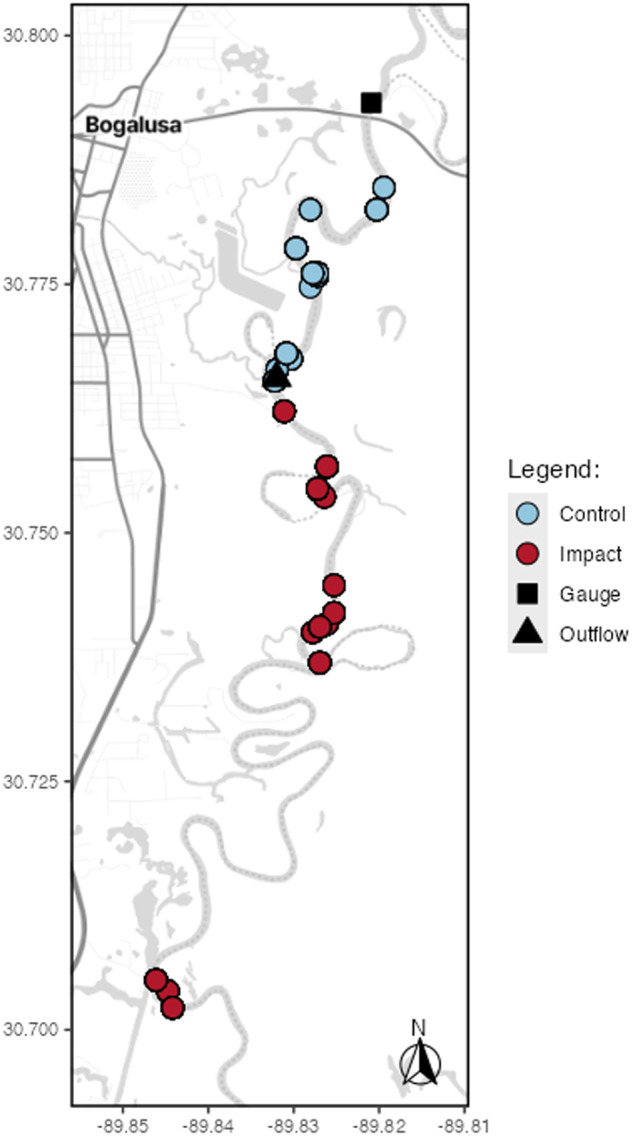
Map of sampling sites upstream (control; light blue circles) and downstream (impact; dark red circles) from pulp mill outfall (triangle). The USGS gauge station (square) from which environmental parameters were sampled is near the town of Bogalusa, LA, USA. Figure 1 long description.

Specimens were sourced from the Royal D. Suttkus Fish Collection at the TUBRI in New Orleans, Louisiana, USA. Fish were collected by Suttkus and colleagues between 1963 and 2005 through seining upstream (northern limit: 30°46′57.0″ N 89°49′13.0″ W) and downstream (southern limit: 30°42′08.0″ N 89°50′39.0″ W) of a pulp-mill outfall (Fig. 1). The fish were fixed in formalin and stored in jars of ethanol. Each jar contained individuals from 1 fish species, collected at a particular site and date.

Seven common Pearl River fish species representing different trophic levels were selected for dissection, including the highfin carpsucker *Carpiodes velifer,* the mosquitofish *Gambusia affinis*, the Mississippi silvery minnow *Hybognathus nuchalis,* the channel catfish *Ictalurus punctatus,* the emerald shiner *Notropis atherinoides,* the saddleback darter *Percina vigil* and the bullhead minnow *Pimephales vigilax*. These fish species were selected based on the availability of samples that allowed us to have a balanced sample size across time and space. Specifically, we selected lots that fell within the geographical boundaries of our study site and time frame. We aimed to dissect 20 individuals per fish species (7 species in total) in each of 2 treatment categories (control [upstream] versus impact [downstream]), in each of 4 decades (1963–2005). Only lots (i.e. jars) where fish were sufficiently large (>30 mm total length) and abundant (>4 fish per lot) were selected. Lots were selected for inclusion by stratified random sampling, aiming for 5 lots per location–decade combinations (e.g. upstream_1964–1973). For each lot, we used a random number generator to randomly select 2 or 4 individuals per jar, depending on the fish species (Table 1). We ensured that their size (i.e. total length) fell within a specific size range: large enough to be dissected and within a size range that encompassed the average size range across the entire time series. When an individual fish did not fit the size specification, the selection process was repeated to obtain a different fish. Finally, after each individual fish was selected, the information associated with the jar was recorded (i.e. catalogue number, the total number of fish in the jar, date and site of collection including latitude and longitude, a description of the locality and the initials of the collector; Table S1), the fish was photographed, weighed, measured (total length and standard length in mm) and placed in a petri dish with 70% ethanol for dissection and parasite inspection.

**Table 1.** Size range of individuals dissected per fish species Table 1 long description.

Fish species	Trophic level^a^ mean ± SE	Total length (mm) mean (min–max)	*n* per jar	*n* dissected
*Carpiodes velifer*	2.3 ± 0.1	45.1 (29–85)	2	180
*Gambusia affinis*	3.1 ± 0.2	33.7 (20–53)	4	208
*Hybognathus nuchalis*	2.0 ± 0.0^b^	66.9 (33–121)	4	221
*Ictalurus punctatus*	4.2 ± 0.3	80.0 (31–139)	4	88
*Notropis atherinoides*	2.8 ± 0.3	58.6 (35–98)	4	206
*Percina vigil*	3.3 ± 0.4	44.7 (30–59)	2	193
*Pimephales vigilax*	2.6 ± 0.1	56.7 (26–96)	4	92

a www.fishbase.org.

b for *Hybognathus* spp.

### Parasitological dissections

We used a semi-destructive parasitological dissection method in which we dissected only the right side of the fish and the internal organs, leaving the left side untouched and preserving the external morphology of the fish. The right eye, pectoral fin and pelvic fin were extracted and placed in the petri dish together with the body of the fish. The gills from the right side were removed and placed in a separate container in 70% ethanol. The fish was sliced open from the isthmus to the vent and internal organs (i.e. liver, kidney, intestines, gonads and gall bladder) were removed and placed in another petri dish. Parasite detection proceeded by examining the fish externally (skin, fins), then the body and buccal cavities under a stereomicroscope. The eye was macerated with forceps and inspected. The liver, kidney and gonads were inspected visually for pseudocysts while probing with forceps under the stereomicroscope. The intestinal wall was pulled apart, exposing the intestinal content, followed by sorting through the contents with forceps. We photographed the pseudocysts under a stereomicroscope and examined them as a fresh mount under a compound light microscope. Myxozoan spores were photographed and a genus was assigned. These pictures along with the genus and organ of infection were added to a parasite identification guide that was used for the rest of the fish individuals. After encountering pseudocysts in a new individual, at least 1 spore was inspected under the microscope, and the appearance of the spores was compared to our identification guide. The gallbladders of *C. velifer, G. affinis* and *P. vigil* were inspected for myxozoans by rupturing each on a slide as a fresh mount and inspecting it under a light compound microscope at 400×. Since the gallbladder was not always found, an ‘NA’ was recorded for uninspected gall bladders, and a ‘0’ and a ‘1’ were assigned to negative and positive infections, respectively. Photomicrographs of gall-bladder infecting myxozoans were added to our parasite ID guide as described above. The gills were inspected by probing each filament with a thin needle. The remaining liquid from the petri dishes was screened in a grid pattern under the microscope. All parasites were vouchered in 70% ethanol. Digital images of the parasites were captured at 1000× magnification using a compound light microscope, and from these, spores were identified to genus level (Hoffman and Williams, 1999; Lom and Dyková, 2006).

### Abiotic variables

Data on abiotic variables were accessed from a USGS gauge upstream of our sampling sites (30°47′35.7″ N 89°49′15.3″ W). These data were available for the period between 1976 and 1992 and included water temperature, nitrogen concentration and metal concentrations (As, Ba, Cd, Cr, Cu, Pb, Mg, Mn, Hg, Ni, Zn). Abiotic variables were calculated as the average of the driver (temperature and nitrogen and metal concentrations) over the 365 days prior to the host’s collection. Nitrogen concentration (mg-N L^−1^) was used as reported for nitrogen in its mixed forms (NH_3_, NH_4_^+^, organic, NO_2_^-^ and NO_3_^-^).

### Statistical analyses

Statistical analyses were performed with R (v3.6.1; R Core Team, 2021). Myxozoan taxa were referred to and analyzed as genus + host + tissue. To ensure sufficient statistical power, analyses were performed for parasites with a prevalence greater than 5% within their host species across time. Analyses were divided in 2 parts. The first part pertained only to temporal change: Did myxozoan prevalence and abundance change over time (1963–2005)? The second part of the analysis concerned potential drivers of change: (1) Does the pulp mill outfall have an effect on parasite prevalence and abundance?; (2) Do abiotic variables (temperature and nitrogen and metal concentrations) explain change over time in myxozoan prevalence and abundance? For the second question, only upstream (control) samples were used, since environmental data were only available for that region and we could not assume that it would be representative of downstream conditions. Myxozoan prevalence was used as the response variable for taxa infecting the gallbladder, since these myxozoans numbered in the thousands and therefore were not counted. For myxozoans producing pseudocysts, pseudocyst count was used as the response variable. For all analyses, generalized linear mixed models were used and, given the inherent positive relationship between parasite abundance and host size (Poulin, 1999), an offset term was included for host total length.

#### Change over time in the abundance of parasites

To test whether myxozoan prevalence and abundance increased or decreased through time, we used a generalized linear mixed model with the glmmTMB function (Brooks et al., 2017). The response variable was ‘parasite presence’ (for myxozoans infecting the gallbladder; equation (1)) or ‘parasite abundance’ (# of pseudocysts per fish host for myxozoans producing pseudocysts; equation (2)), the fixed predictor was time (i.e. year of collection), and the random structure included site and season. Site was included as a random effect given that some sites were resampled over time. Site categories were designated by visually clustering the location of sampling events. Season was included because fish were collected during different months across the year and myxozoan abundance and prevalence was expected to shift with season. December through February was set as winter, March through May as spring, June through August as summer and September through November as fall. A binomial error distribution was used when including myxozoan presence as response variable (equation (1)) and negative binomial for myxozoan abundance (equation (2)). Models with negative binomial distribution were fit with linear (nbinom1) and quadratic (nbinom2) parameterization, and with log- and square root-link functions for a total of 4 models per myxozoan taxa. The best model was chosen according to the Akaike information criterion and model diagnostics using the DHARMa package (version 0.4.7; Hartig, 2024) including qq-plots, overdispersion, Kolmogorov–Smirnov and outlier tests, and by plotting observed vs predicted residuals. The transformed total length was used as an offset, and the transformation was matched to the link-function (log or square root) associated with the error distribution.

(1) $$amp;Presence_{ijk}\;\sim \;Bernoulli\left(p_{ijk}\right)\mathrm{\backslash nonumber}\;amp;logit\left(p_{ijk}\right)=\;\beta_{0}+\;\beta_{1}*Year_{ijk}+\;Site_{i}+\;Season_{j}+Offset_{ijk}\mathrm{\backslash nonumber}\;amp;Site_{i}\;\sim \;𝒩\left(0,\sigma_{Site}^{2}\right)\mathrm{\backslash nonumber}\;amp;Season_{j}\;\sim \;𝒩\left(0,\sigma_{Season}^{2}\right)$$

where *Presence_ijk_* corresponds to 1 when fish *k* collected at site *i* during season *j* was infected with the parasite and to 0 when not infected, *Year_ijk_* represents the year of collection, *Offset_ijk_* is the log-transformed total length (mm) of the fish as offset, and *Site_i_* and *Season_j_* correspond to random intercepts with normal distribution a mean of 0 and variance *σ*^2^*_x_*.

(2) $$amp;Abundance_{ijk}\;\sim \;NegBin\left(\mu_{ijk},\theta \right)\mathrm{\backslash nonumber}\;amp;g\left(\mu_{ijk}\right)=\;\beta_{0}+\beta {\;}_{1}*Year_{ijk}+\;Site_{i}+\;Season_{j}+Offset_{ijk}\mathrm{\backslash nonumber}\;amp;Site_{i}\;\sim \;𝒩\left(0,\sigma_{Site}^{2}\right)\mathrm{\backslash nonumber}\;amp;Season_{j}\;\sim \;𝒩\left(0,\sigma_{Season}^{2}\right)$$

where *Abundance_ijk_* represents the number of pseudocysts per fish *k* collected at site *i* during season *j, g*() is the link function (log or square root), *Year_ijk_* represents the year of collection, and *Site_i_* and *Season_j_* correspond to random intercepts with normal distribution, mean 0 and variance *σ*^2^*_x_*, and *Offset_ijk_* is the transformed (log or square root depending on the link function) total length of the fish as offset.

#### Assessing pulp mill impact and environmental correlates of change over time in the abundance of parasites

To assess the impact of the pulp mill outfall, equation (1) for prevalence and equation (2) for abundance were used but with site (control [upstream] versus impact [downstream]) as a fixed factor. Since we had access to data only from an upstream gauge, correlations between temperature, metal and nitrogen concentrations, and parasite abundance were performed only for the control (upstream from pulp mill) sites, and for the parasites that had a significant change in abundance over time. To preserve degrees of freedom, we replaced raw metal data with scores from 2 principal components (PCs). The principal component analysis (PCA) was performed using the missMDA package (Josse and Husson, 2016). The number of dimensions for the PCA was estimated using the *estim_ncpPCA()* function, and missing values were imputed using the *imputePCA()* function. The first 2 PCs were used in downstream analyses, explaining a total of 62.61% of the variance. The resulting first PC (PC1) was associated with high concentrations of Ba, Pb, Mn and Ni with contributions of 16.3%, 10.0%, 19.4% and 12.2%, respectively, while the second PC (PC2) was mainly associated with high concentrations of Cr with a contribution of 26% to this dimension. To evaluate the impact of temperature, nitrogen and metal concentration on parasite abundance, we also used the glmmTMB package. We used the same response variables and offset for host total length as in the temporal analyses (equation (3)). The fixed predictors were temperature and its interaction with nitrogen concentration and the PCs for the metal concentrations (equation (3)). The random structure included site and season.

(3) $$amp;Abundance_{ijk}\;\sim \;NegBin\left(\mu_{ijk},\theta \right)\mathrm{\backslash nonumber}\;amp;g\left(\mu_{ijk}\right)=\beta {\;}_{0}+\;\beta_{1}\cdot Temp_{ijk}+\;\beta_{2}\cdot N_{ijk}+\;\beta_{3}\cdot E1_{ijk}+\beta {\;}_{4}\cdot E2_{ijk}\mathrm{\backslash nonumber}\;amp;\;\;+\beta_{5}\cdot Temp_{ijk}\times N_{ijk}+\beta_{6}\cdot Temp_{ijk}\times E1_{ijk}\mathrm{\backslash nonumber}\;amp;\;\;+\beta_{7}\cdot Temp_{ijk}\times E2_{ijk}+\;\;Site_{i}+\;Season_{j}+Offset_{ijk}\mathrm{\backslash nonumber}\;amp;Site_{i}\;\sim \;𝒩\left(0,\sigma_{Site}^{2}\right)\mathrm{\backslash nonumber}\;amp;Season_{j}\;\sim \;𝒩\left(0,\sigma_{Season}^{2}\right)$$

where *Temp_ijk_* and *N_ijk_* represent the scaled mean water temperature and mean nitrogen concentration (mg-N L^−1^) over 365 days prior collection of fish *k*, and *E1_ijk_* and *E2_ijk_* correspond to the PC1 and PC2 of metal concentrations. The rest of the terms correspond to those in equation (2).

## Results

### Parasite prevalence and abundance

**Figure 2.**
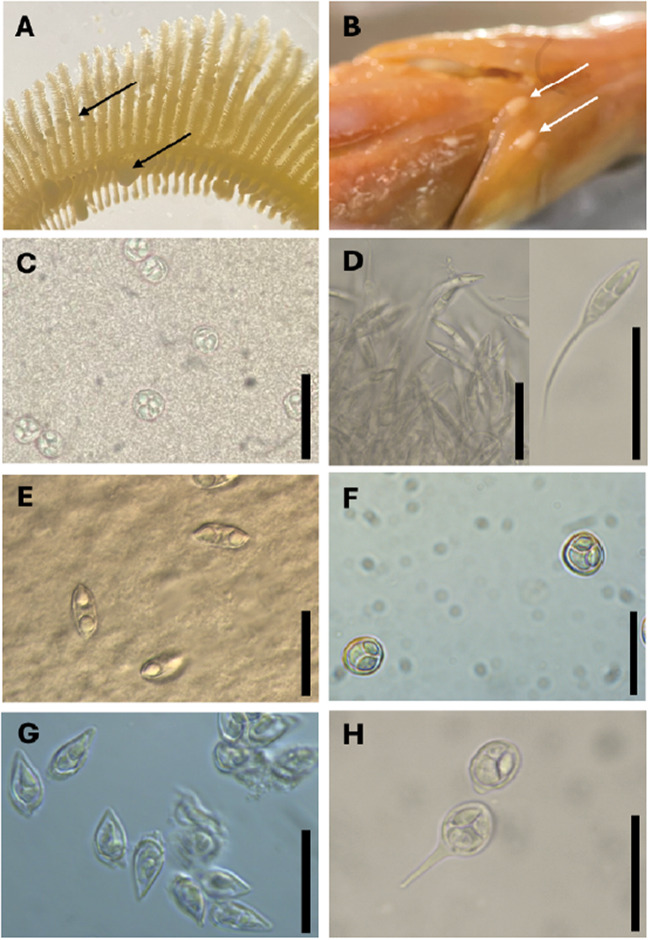
Photomicrographs of myxozoan infections in formalin-fixed museum fish: Pseudocysts (arrowed) on (A) the gills of *Carpiodes velifer* infected with *Myxobolus* and (B) the skin of *Hybognathus nuchalis* infected with *Unicauda*. Examples of myxospores encountered: (C) *Chloromyxum* from *C. velifer*, (D) a cluster of *Henneguya* (D-left) and an isolated spore (D-right) from *Ictalurus punctatus*, (E) *Myxidium* from *H. nuchalis*, (F) *Myxobolus* from *C. velifer*, (G) *Thelohanellus* from *Pimephales vigilax* and (H) *Unicauda* from *H. nuchalis.* Scale bars = 20 µm. Figure 2 long description.

Myxozoans were recovered from 6 of the 7 fish species dissected. The myxozoans represented 6 genera: *Chloromyxum, Henneguya, Myxidium, Myxobolus, Thelohanellus* and *Unicauda. Percina vigil* was the only fish in which no myxozoans were found. *Carpiodes velifer* had the highest prevalence of infection across time with 71% infected with *Myxobolus* (75% in control and 67% in impact; Table 2), and 15% infected with *Chloromyxum* (8.7% and 13.6% for control and impact, respectively; Table 2). *Myxobolus* pseudocysts were detected the gill (Fig. 2A), eye, kidney, skin and the anal, caudal, dorsal, pectoral and pelvic fins (Table 2), while *Chloromyxum* infections were found only in the gall bladder. *Notropis atherinoides* was found to be infected uniquely with *Myxobolus* (19% prevalence, 19.2% and 18.8% for control and impact, respectively; Table 2) in gill and skin tissue, while the caudal and dorsal fins of *Ictalurus punctatus* individuals were infected with *Henneguya* (prevalence 7%; Table 2). Thirteen percent of *Pimephales vigilax* were infected with *Thelohanellus*, in skin and occasionally the caudal, dorsal and pectoral fins, while 12% were infected with *Myxobolus* in connective tissue, gills, gonads and kidney. *Hybognathus nuchalis* harbored *Myxobolus* (6% prevalence; control: 4.0%, impact: 8.3%) with pseudocysts detected in the eye, caudal fin and gill, *Unicauda* (3% prevalence) in the skin (Fig. 2B) and *Myxidium* (2% prevalence) uniquely in the gall bladder. Lastly, *Gambusia affinis* was infected with *Myxidium* in the gall bladder and *Myxobolus* in the gills (11% and 2%, respectively). In terms of abundance, the group ‘*Myxobolus + Carpiodes velifer +* gills’ were highest (average 80 pseudocysts/fish; range 0–1118 pseudocysts/fish; 85 pseudocysts/fish and 83 pseudocysts/fish for the control and impact sites, respectively). The rest of the pseudocyst-producing myxozoans had a mean abundance of fewer than 6 pseudocysts per fish (Table 2). Spores appeared to be in good condition (Fig. 2C-H). For higher granularity in the changes of prevalence and abundance at different points of time refer to the supplementary material (Figure S1 and Figure S2).

**Table 2.** Prevalence and mean abundance of myxozoans from fish collected upstream and downstream a paper pulp mill Table 2 long description.

Parasite genus	Fish species	Organs of infection	Site	*n* dissected fish	Prevalence (%)	Mean abundance (# pseudocysts/fish)
*Chloromyxum*	*Carpiodes velifer*	Gall bladder	Control	92	8.7	
			Impact	88	13.6	
*Henneguya*	*Ictalurus punctatus*	Caudal fin, dorsal fin	Control	31	9.7	0.13
			Impact	57	5.3	5.70
*Myxidium*	*Gambusia affinis*	Gall bladder	Control	84	6.0	
			Impact	124	9.7	
	*Hybognathus nuchalis*	Gall bladder	Control	100	4.0	
			Impact	121	0.8	
*Myxobolus*	*C. velifer*	Eye, gill, kidney, skin and anal, caudal, dorsal, pectoral and pelvic fins	Control	92	75.0	85.37
			Impact	88	67.0	83.02
	*G. affinis*	Gill	Control	84	2.4	0.02
			Impact	124	1.6	0.02
	*H. nuchalis*	Eye, caudal fin, gill	Control	100	4.0	0.08
			Impact	121	8.3	0.38
	*Notropis atherinoides*	Gill, skin	Control	78	19.2	1.44
			Impact	128	18.8	3.63
	*Pimephales vigilax*	Connective tissue, gill, gonad, kidney	Control	88	14.8	1.14
			Impact	105	9.5	1.74
*Thelohanellus*	*P. vigilax*	Skin and caudal, dorsal and pectoral fins	Control	88	12.5	0.41
			Impact	105	12.4	0.78
*Unicauda*	*H. nuchalis*	Skin, gills	Control	100	4.0	0.58
			Impact	121	2.5	0.17

*Percina vigil* (*n* = 92) was omitted since no myxozoans were detected in this fish at either site.

### Effect of time on prevalence and abundance

**Figure 3.**
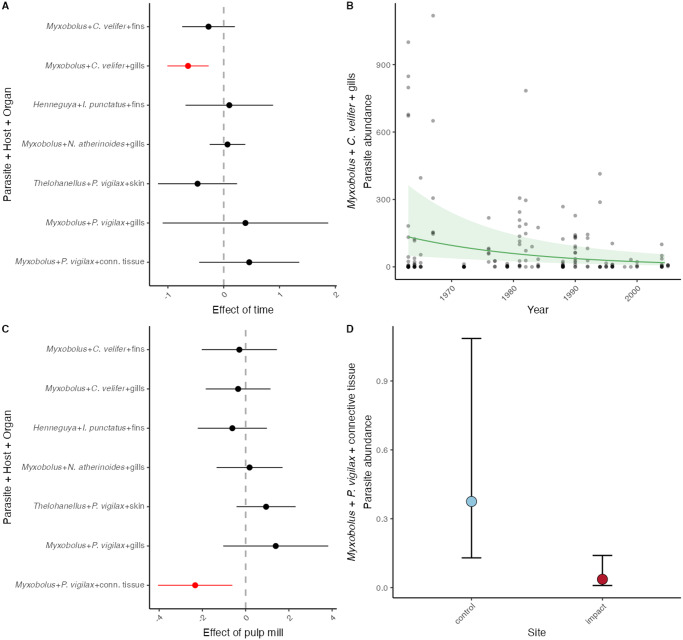
(A) Change in myxozoan pseudo-abundance from 1963 to 2005, for parasite–host–organ combinations and (B) for ‘*Myxobolus* + *C. velifer +* gills’ across sites, and (C) effect of pulp mill outfall on parasite abundance relative to the control sites and (D) predicted abundance of *Myxobolus* infecting *P. vigilax* connective tissue for control (light blue) and impact (dark red) sites (upstream and downstream from pulp mill, respectively). Estimates are based on generalized linear mixed models with negative binomial distribution. Estimates in red represent significant effects of time (A) and pulp mill outfall (C). Figure 3 long description.

Based on estimates from the generalized linear mixed models (Fig. 3A), only ‘*Myxobolus* + *C. velifer +* gills’ exhibited a significant change over time, with a decrease in abundance of about 86% from 1963 to 2005 (scaled estimate = −0.639, CI = −1.009 to −0.270, *p* < 0.001; Fig. 3B). The rest of the myxozoans did not have a significant change in abundance or prevalence over time (Fig. 3A). In the case of pseudocyst-producing myxozoans, a non-significant decrease over time was observed for ‘*Myxobolus + C. velifer +* fins’ (scaled estimate = −0.272, CI = −0.272 to 0.200, *p* = 0.258), and for ‘*Thelohanellus + P. vigilax* + gills’ (scaled estimate = −0.469, CI = −1.177 to 0.239, *p* = 0.194). A non-significant increase over time was observed for ‘*Henneguya* + *I. punctatus* + gills’ (scaled estimate = 0.100, CI = −0.684 to 0.885, *p* = 0.802), ‘*Myxobolus* + *N. atherinoides* + fins’ (scaled estimate = 0.067, CI = −0.251 to 0.386, *p* = 0.679), ‘*Myxobolus* + *P. vigilax* + gills’ (scaled estimate = 0.457, CI = −0.440 to 1.353, *p* = 0.318) and ‘*Myxobolus* + *P. vigilax* + fins’ (scaled estimate = 0.389, CI = −1.094 to 1.872, *p* = 0.607). From those myxozoans infecting the gallbladder, no significant change over time was observed in prevalence of infection. However, *Chloromyxum* infecting *C. velifer* experienced a non-significant decline over time (scaled estimate = −0.275, CI = −0.738 to 0.188, *p* = 0.244) and *Myxidium* infecting *G. affinis* experienced a non-significant increase over time (scaled estimate = 0.240, CI = −0.276 to 0.757, *p* = 0.362).

### Effect of pulp-mill outfall and abiotic variables on abundance

**Figure 4.**
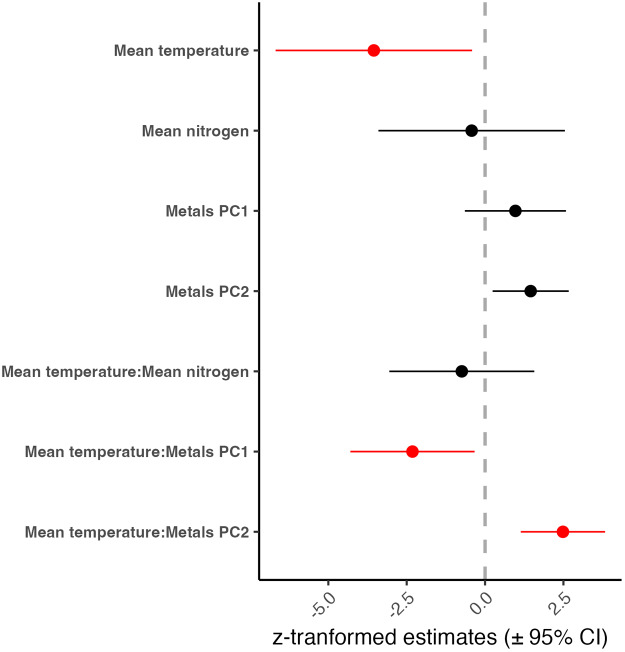
Estimates (scaled and centred) for the effect of each predictor and their interaction with temperature on parasite abundance for ‘*Myxobolus* + *C. velifer +* gills’ based on generalized linear mixed models with negative binomial distribution. Estimates in red represent significant terms. For this analysis, only sites upstream from the pulp mill (near the gauge) were included. Figure 4 long description.

**Figure 5.**
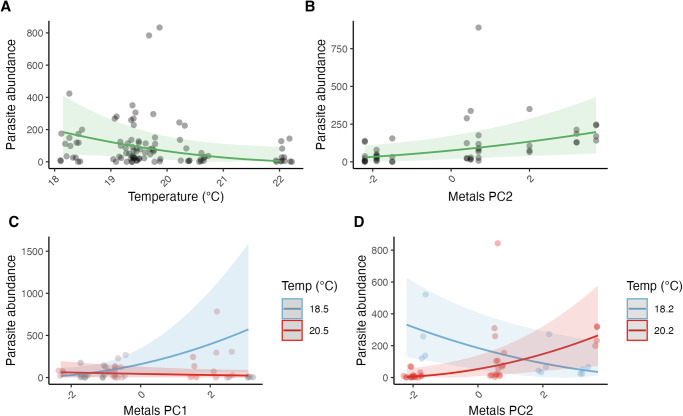
(A) Main effect of temperature and (B) the second metal principal component on parasite abundance (# pseudocysts/fish) of ‘*Myxobolus* + *C. velifer +* gills’, and the interaction between temperature and (C) the first and (D) second principal components of metals based on a generalized linear mixed model with negative binomial distribution. For this analysis, only sites upstream from the pulp mill (near the gauge) were included. Figure 5 long description.

Based on the GLMM with negative binomial error distribution, only *Myxobolus* infecting the connective tissue of *P. vigilax* exhibited lower abundance in the impacted site compared to the control (estimate = −2.3341, *p* < 0.01; Fig. 3C-D). To investigate the potential causes of the significant temporal decline of ‘*Myxobolus* + *C. velifer +* gills’, we evaluated the impact of temperature and its interaction with nutrients and metals over time. In the region, water temperature increased from an average of 18.9 °C (min: 4.0 °C, max: 29.0 °C) in 1968 to 21.3 °C (min: 7.5 °C, max: 29.0 °C) in 1994 (see supplementary Figure S3). Concentration of metals Ba, Cd, Fe, Mn and Zn, declined over the years, while Mg increased (see supplementary Figure S4). Based on generalized linear mixed models (Fig. 4), temperature had a significant negative effect on parasite abundance (scaled estimate = −3.548, CI = −6.68 to −0.414, *p* = 0.03; Fig. 5A) while the PC2 for the metals (associated with high concentrations of Cr) had a positive effect on abundance (scaled estimate = 1.454, CI = 0.240–2.670, *p* = 0.02; Fig. 5B). Temperature also had an interactive effect with both metal PCs (Fig. 5C, D). For PC1 (associated with Ba, Pb, Mn and Ni), the association with parasite abundance diminished from positive to zero with increasing temperature. For PC2 (associated with Cr), the association with parasite abundance flipped from negative to positive with increasing temperature.

## Discussion

To our knowledge, this is the first study to evaluate the impact of stressor interactions on myxozoan abundance. Museum collections allowed us to characterize ∼40 years of myxozoan abundance data, including 6 genera across 6 fish species. We tracked changes across time and identified a significant decrease in abundance for ‘*Myxobolus* + *C. velifer +* gills’. We also found a decline in abundance of *Myxobolus* infecting the connective tissue of *P. vigilax* downstream of the pulp mill outfall, and a correlation between *Myxobolus* abundance and the main effect of temperature and metal concentrations, as well as stressor interactions (temperature and metal concentrations). Moreover, we report new myxozoan infections in multiple fish species.

Although the focus of this study is not on taxonomy, the museum collection gave us access to diverse myxozoans. After dissecting 1188 fishes across 7 species, we found 6 different myxozoan genera: *Chloromyxum, Henneguya, Myxobolus, Myxidium, Thelohanellus* and *Unicauda*; with *Myxobolus* being found most commonly. *Carpiodes velifer* had the highest prevalence of infection and *Hybognathus nuchalis* harbored 3 different myxozoan genera. The higher prevalence and diversity of myxozoans in *C. velifer* and *H. nuchalis,* respectively, might be attributed to their feeding behaviour. *C. velifer* feeds mostly on benthic invertebrates including annelids, grubbed from sediments, while *H. nuchalis* mainly feeds on organic matter and algae from bottom ooze (Lee et al., 1980; Spiegel et al., 2011). Previously, *C. velifer* was reported to be infected with *M. obliquus* (Hoffman and Williams, 1999), *M. ovalis* (Hoffman and Williams, 1999) and *M. discrepans* (Lom and Cone, 1996). We also found *Myxobolus* and added *Chloromyxum* infecting the gall bladder of this host. Similarly, *Hybognathus nuchalis* is known to host *Unicauda macrura* (Gurley, 1893). In this study, *H. nuchalis* was found to host *Unicauda*, and we additionally found *Myxidium* and *Myxobolus*, both previously unreported. *Ictalurus punctatus* is known to host 9 species of *Henneguya* (*H. adiposa, H. diversis, H. exilis, H. limatula, H. longicauda, H. postexilis, H. sutherlandi, H. pelis* and *H. mississippiensis*, Minchew, 1977; Hoffman and Williams, 1999; Eiras and Adriano, 2012; Rosser et al., 2015), 2 species of *Myxidium* (*My. bellum* and *My. macrocapsulare)* (Hoffman and Williams, 1999) and *Sphaerospora ictaluri* (Hoffman and Williams, 1999). In this study, we detected only *Henneguya* infections. In the case of *Notropis atherinoides*, Hoffman and Williams (1999) report *Chloromyxum* sp., *Dicauda atherinoides, Myxidium* sp., *Myxobolus* sp. and *Unicauda* sp. from this host, while Horner et al. (2010) also report *Myxobolus notropis* infections. The *N. atherinoides* individuals we examined were infected only with *Myxobolus.* The mosquito fish *Gambusia affinis* is known to host *Myxobolus pharyngeus* and *Henneguya gambusi* (Hoffman and Williams, 1999), and we found *G. affinis* to host *Myxobolus* and a novel *Myxidium*. We found *Pimephales vigilax* infected with *Myxobolus* and *Thelohanellus* species. In the literature, this fish has been found to host *Thelohanellus notatus* and *Myxobolus angustus* (Hoffman and Williams, 1999). Finally, *Percina vigil* was the only fish without any myxozoans, in concordance with the lack of prior literature reports.

*Myxobolus* infecting the connective tissue of *P. vigilax* declined in abundance just downstream of the pulp mill outfall. Identifying a specific driver for this decline was not possible due to the lack of environmental data downstream of the pulp mill. However, the analysis based on the upstream samples suggests that metal concentration, temperature or nutrients do not correlate with changes in the abundance of *Myxobolus* in the connective tissue of *P. vigilax*. Therefore, it is likely that this decrease in myxozoan abundance is an indirect outfall effect driven by changes in benthic community composition. Pulp mill outfalls are associated with changes in the community composition of benthic invertebrates with increased abundance of annelids and chironomid larvae (Harris et al., 1992; Sibley et al., 1997). *Pimephales vigilax* is an opportunistic feeder, feeding on insect larvae, especially chironomid larvae, or organic matter depending on availability (Parker, 1964; Whitaker, 1977). With increased abundance of chironomid larvae, *P. vigilax* might shift its diet to its preferred prey. While focusing on chironomid larvae, organic matter and associated organisms (i.e. annelids) become less attractive as a food source thus decreasing *P. vigilax* exposure to myxozoans. Since we did not have historical data on benthic community composition, this explanation remains speculative. However, the differential response of host–parasite combinations to pulp mill outfall highlights the importance of interspecific ecological interactions as an important player behind changes in parasite diversity.

We identified a significant main effect of temperature and metal concentrations, and an interactive effect between temperature and metal concentrations on parasite abundance. The direction of the main effect of metal concentrations was positive, while the effect of temperature was negative, contrary to what we expected. Regarding metals, only 1 study has examined effects of metals on myxozoan infections, evaluating specifically the combined effect of Cd and a myxozoan infection on annelid host survival (Shirakashi and El-Matbouli, 2010). Chronic exposure to Cd (0.01–2 mg L^−1^) promoted the production of myxozoan spores, while myxozoan-infected oligochaetes survived longer than uninfected ones after a 72 h exposure to Cd (LC_50-uninfected_ 0.05 ± 0.04 mg L^−1^; LC_50-infected_ 0.21 ± 0.21 mg L^−1^) (Shirakashi and El-Matbouli, 2010). The increase in spore production in response to Cd found by Shirakashi and El-Matbouli (2010) might be aligned with the positive correlation between myxozoan abundance and metal concentration found in our study. If more spores are produced by the annelids in response to metals, the risk of transmission to fish increases resulting in higher myxozoan abundance. However, the mechanisms behind higher spore production in response to Cd are still unresolved (Shirakashi and El-Matbouli, 2010). Generally, an increase in myxozoan abundance is expected with increasing temperature, due to accelerated spore replication, development and transmission (Okamura et al., 2011; Strepparava et al., 2018; Bolin et al., 2024). This relationship was not apparent from our results, possibly because non-pathogenic myxozoans respond differently to temperature than the well-studied pathogenic myxozoans like *Ceratonova shasta* (causative agent of salmonid enteronecrosis (Ray et al., 2012) and *T. bryosalmonae* (causative agent of proliferative kidney disease (Okamura et al., 2011). In *C. shasta*, transmission can occur at temperatures as low as 4 °C (Ching and Munday, 1984), while host mortality risk increases with rising temperatures (Ray et al., 2012). The same has been observed for *T. bryosalmonae,* whose pathology is exacerbated at warmer temperatures (Bettge et al., 2009).

Myxozoan prevalence is most responsive to temperature in areas of high thermal variability and strong seasonal thermal changes (Bolin et al., 2024). The Pearl River experiences episodic heat waves, with temperatures reaching 31.5 °C in summer. While the thermal performance of ‘*Myxobolus* + *C. velifer +* gills’ is unknown, it is plausible that these high temperatures might exceed the optimal range for the parasite, or the hosts involved in the life cycle. The interaction between temperature and metal concentrations was expected. It is known that temperature modulates toxicodynamics by enhancing bioavailability, uptake and toxicity, while also facilitating elimination and metabolic responses (Noyes et al., 2009; Hooper et al., 2013). The strongest interaction we found suggests that, at warmer temperatures, metal concentrations have a positive effect on parasite abundance. This effect was antagonized at colder temperatures, where a negative correlation between parasite abundance and metal concentration was found. In freshwater fish, metal absorption through the gills is highly important given that it is the main organ used for osmoregulation (Sures et al., 2025). At warmer temperatures fish have increased metabolism, higher respiration and therefore more contact with metals dissolved in the water (Noyes et al., 2009; Hu et al., 2024). On the one hand, this relationship might be beneficial for the parasite (in this case the gill-infecting *Myxobolus*) given that the fish experiences temperature-exacerbated toxic stress, possibly degrading immune function. On the other hand, this increased influx of metals could also be detrimental for the parasite by exposing it to direct stress from the metals. Elucidating stressor interactions in nature is challenging and requires experimental manipulation to establish specific mechanisms. However, with this dataset, we allowed nature to speak for itself, to show us how the interaction between these 2 abiotic parameters shapes parasite abundances in the wild.

With this dataset we assessed how myxozoan abundance changed in its fish host. However, myxozoans have complex life cycles, with benthic annelids being essential for producing the infectious stages to fish (Atkinson et al., 2018; Alama-Bermejo and Holzer, 2021). This component is often missing in life cycles, given that annelids are time consuming to inspect for parasites due to their low prevalence of infection, often as low as 1% (Alexander et al., 2015). Also, both the annelids themselves and those that are infected with myxozoans have patchy distributions, which makes finding myxozoans in these hosts much like finding a needle in a haystack (Alexander et al., 2015). Like fishes, annelids are sensitive to environmental changes (Aston, 1973), which should influence the overall abundance of their parasites. In eutrophic waters, it is expected that myxozoan abundance should increase, given that annelid populations increase in density (Marcogliese and Cone, 2001, 2021; Kaeser et al., 2006; Marcogliese et al., 2009). Since the Pearl River experiences high volume nitrogen-rich discharge from poultry and paper mills, we expected to see a relationship between nitrogen and myxozoan abundance. Such a relationship was not found in this study. While literature suggests that myxozoans might benefit from a nutrient-driven increase in annelid populations (e.g. *Mybolus cerebralis* and its polychaete host; Zendt and Bergersen, 2000), this is not necessarily true for all myxozoan species and their invertebrate hosts. The direction of nutrient effects on annelid populations might be species-specific: other annelid hosts could experience decline in populations from nutrient discharges or no changes at all. For this reason, while giving attention to problematic parasites is important, it is of equal importance to study less burdensome ones. These understudied species could be either facing abundance declines and, in some cases, even extinctions driven by anthropogenic impacts, or they may simply not respond to such impacts due to their and their hosts’ tolerance to changes in abiotic variables (Wood, 2025). Given the importance of benthic annelids in myxozoan life cycles, future studies should consider the impact of stressors in their populations as additional drivers of change. However, in the context of museum collections this is challenging, because benthic annelids are poorly represented relative to fish.

While using museum collections for the study of myxozoan abundances over time is valuable, it has some limitations. Particularly for myxozoans, it is challenging to reach ecological conclusions given that only half of the life cycle is represented. As mentioned above, environmental changes will affect benthic annelid populations, which will inevitably manifest in changes across the whole myxozoan life cycle. Another limitation is that the capture method will affect what species of fish are represented in collections, including only certain size classes (Portt et al., 2006). For instance, in the present study, *Carpiodes velifer* had the highest prevalence of myxozoan infection. *C. velifer* grows very large in the wild, but the method used for its capture (i.e. seining) is biased towards smaller fish. Although the effect of host-age on myxozoan success requires further investigation, young fish, particularly young-of-the-year, are more susceptible to myxozoan infections (Bailey et al., 2021). Therefore, we have a size and age bias in our data given that we mostly sampled the young *C. velifer* available in the museum collection. Another fish species that is susceptible to size and age bias is the channel catfish *Ictalurus punctatus*, which is represented generally by young fish seined from the population. However, the rest of the fishes studied herein reach only small sizes as adults and therefore, older ages are better represented in the museum collection. Another limitation of our study was that we could not perform histology and squashes of all organs due to time and technical constraints. We expect that this led to underestimation of the myxozoan diversity in the Pearl River, given that it is known that diverse myxozoan species exist with high specificity to particular host tissues (i.e. tissue tropism). An additional constraint is that museum tissue is affected by destruction of DNA by formalin fixation, which limits the possibility for DNA amplification, which would otherwise have increased our capacity for myxozoan detection. While DNA amplification of formalin-preserved DNA is possible (Hykin et al., 2015; Hahn et al., 2022), this is challenging for parasites due to the generally limited available parasite biomass relative to the host. Despite these limitations, our results still offer useful insights, because they allowed us to track historical changes in myxozoan abundance in fish hosts and, together with data on abiotic variables, we were able to detect potential drivers behind this change.

The response of parasite abundance to multiple stressors is complex. The myxozoans studied herein responded differently to abiotic variables that not only interact with each other but are expected to change in magnitude in coming decades. With *Carpiodes velifer*-infecting *Myxobolus* significantly decreasing in abundance and other groups having high variability in their abundance throughout time, we expect myxozoan communities to change in ways that we are only beginning to understand. This, together with the many first records of infections found in this study, highlights that museum collections present a unique opportunity to continue investigating how parasite abundance will change over time and the drivers responsible for this change. Therefore, we encourage the further utilization of such collections for this purpose, especially in the context of multiple stressor research.

## Supporting information

10.1017/S0031182026101917.sm001 Díaz-Morales et al. supplementary material Díaz-Morales et al. supplementary material

## Data Availability

The data and code associated with this manuscript are available at Github (https://github.com/wood-lab/TUBRI.git; path = Manuscripts/Myxozoans).

## Supplementary material

The supplementary material for this article can be found at https://doi.org/10.1017/S0031182026101917.

## Figure 1 Long description

The map displays sampling sites near Bogalusa, Louisiana, USA, with specific locations marked by symbols. Control sites are indicated by light blue circles, primarily located upstream. Impact sites are marked by dark red circles, found downstream from the pulp mill outfall. The pulp mill outfall is represented by a triangle, situated centrally on the map. A square denotes the USGS gauge station, located near Bogalusa. The map includes geographic coordinates along the sides, with latitude ranging from approximately 30.700 to 30.800 and longitude from -89.85 to -89.81. The legend clearly defines each symbol's meaning, aiding in the identification of site types and their spatial distribution.

Navigate back to Figure 1.

## Figure 2 Long description

The image A showing a curved band of closely spaced, pale projections arranged in parallel rows on a light background. Two black arrows point toward the projections. The label “A” is at the top left. The image B showing an orange-brown surface with two white arrows pointing toward a narrow, darker line near the center-right. The label “B” is at the top left. The image C showing a pale gray field with multiple small circular forms scattered across the frame. A vertical black scale bar is at the right edge. The label “C” is at the top left. The image D showing several elongated, curved, translucent forms on a gray background. Two vertical black scale bars are at the right side, separated by a gap. The label “D” is at the top left. The image E showing several oval, translucent forms on a tan background. A vertical black scale bar is at the right edge. The label “E” is at the top left. The image F showing a pale blue field with many small circular dots and two larger circular forms with ring-like outlines. A vertical black scale bar is at the right edge. The label “F” is at the top left. The image G showing multiple teardrop-shaped translucent forms on a blue background, with additional clustered shapes near the upper right. A vertical black scale bar is at the right edge. The label “G” is at the top left. The image H showing two round translucent forms, each connected to a narrow elongated extension. A vertical black scale bar is at the right edge. The label “H” is at the top left.

Navigate back to Figure 2.

## Figure 3 Long description

The image A showing a dot and whisker plot with the vertical axis labeled “Parasite plus Host plus Organ” and the horizontal axis labeled “Effect of time.” The horizontal axis tick labels are “negative 2,” “negative 1,” “0,” “1,” and “2,” with a vertical dashed reference line at “0.” Seven labeled rows each show one circular point with a horizontal whisker: “Myxobolus plus C. velifer plus fins,” “Myxobolus plus C. velifer plus gills,” “Henneguya plus I. punctatus plus fins,” “Myxobolus plus N. atherinoides plus gills,” “Thelohanellus plus P. vigilax plus skin,” “Myxobolus plus P. vigilax plus gills,” and “Myxobolus plus P. vigilax plus con. tissue.” The plotted points appear on both sides of the “0” line, with several points positioned to the positive side and at least one point positioned to the negative side. The image B showing a scatter plot with the horizontal axis labeled “Year” and the vertical axis labeled “Parasite abundance.” The vertical axis tick labels are “0,” “300,” “600,” and “900.” The horizontal axis tick labels are “1960,” “1970,” “1980,” “1990,” and “2000.” The y-axis label area includes the text “Myxobolus plus C. velifer plus gills.” Many points are clustered near the baseline close to “0,” with additional points extending upward, including points above “900.” A smooth curve with a shaded band is drawn across the scatter points. The image C showing a dot and whisker plot with the vertical axis labeled “Parasite plus Host plus Organ” and the horizontal axis labeled “Effect of pulp mill.” The horizontal axis tick labels are “negative 2,” “negative 1,” “0,” “1,” and “2,” with a vertical dashed reference line at “0.” Seven labeled rows each show one circular point with a horizontal whisker: “Myxobolus plus C. velifer plus fins,” “Myxobolus plus C. velifer plus gills,” “Henneguya plus I. punctatus plus fins,” “Myxobolus plus N. atherinoides plus gills,” “Thelohanellus plus P. vigilax plus skin,” “Myxobolus plus P. vigilax plus gills,” and “Myxobolus plus P. vigilax plus con. tissue.” The plotted points appear on both sides of the “0” line, with multiple points positioned to the positive side and at least one point positioned to the negative side. The image D showing a point and whisker plot with the horizontal axis labeled “Site” and the vertical axis labeled “Parasite abundance.” The vertical axis tick labels are “0.0,” “0.3,” “0.6,” and “0.9.” The x-axis categories are “control” and “impact.” Each category has one circular point with a vertical whisker. The “control” point is plotted higher than the “impact” point and the “control” whisker spans a larger vertical range than the “impact” whisker. Across the four images, the plots present effect estimates relative to a “0” reference line (images A and C), a year-by-year scatter of parasite abundance with a fitted curve (image B) and a two-category site comparison of parasite abundance (image D).

Navigate back to Figure 3.

## Figure 4 Long description

The plot displays z-transformed estimates (plus or minus 95 percent CI) for various predictors and their interactions with temperature on parasite abundance. The y-axis lists predictors: Mean temperature, Mean nitrogen, Metals PC1, Metals PC2, Mean temperature:Mean nitrogen, Mean temperature:Metals PC1 and Mean temperature:Metals PC2. The x-axis is labeled z-transformed estimates (plus or minus 95 percent CI) with tick marks at 0, 2.5 and 5. Each predictor is represented by a dot with a horizontal line indicating the confidence interval. Mean temperature and Mean temperature:Metals PC2 are highlighted in red, indicating significant terms. Mean temperature shows a negative estimate crossing zero, while Mean temperature:Metals PC2 has a negative estimate not crossing zero, indicating a significant negative effect. Metals PC1 and Metals PC2 have positive estimates, with confidence intervals crossing zero, suggesting non-significant effects. Mean nitrogen and Mean temperature:Mean nitrogen show positive estimates with confidence intervals crossing zero, indicating non-significant effects. The vertical dashed line at 0 serves as a reference for distinguishing positive and negative effects.

Navigate back to Figure 4.

## Figure 5 Long description

A, B, C, D. The image A showing a scatter plot with a fitted curve and a shaded band. The x-axis label is Temperature (degrees Celsius), with tick labels 16, 18, 20, 22. The y-axis label is Parasite abundance, with tick labels 0, 200, 400, 600, 800. Points are spread across the plot, including points near 800. The fitted curve slopes downward from left to right. The image B showing a scatter plot with a fitted curve and a shaded band. The x-axis label is Metals PC2, with tick labels minus 2, 0, 2. The y-axis label is Parasite abundance, with tick labels 0, 250, 500, 750. Points include a point near 800. The fitted curve slopes upward from left to right. The image C showing a scatter plot with two fitted curves and two shaded bands, plus a legend. The x-axis label is Metals PC1, with tick labels minus 2, 0, 2. The y-axis label is Parasite abundance, with tick labels 0, 500, 1000, 1500. The legend title is Temp (degrees Celsius), with two entries labeled 18.5 and 20.5. One fitted curve rises from left to right and reaches above 500 near Metals PC1 around 2, with a wide shaded band. The other fitted curve stays near 0 across the x-axis range with a narrow shaded band. The image D showing a scatter plot with two fitted curves and two shaded bands, plus a legend. The x-axis label is Metals PC2, with tick labels minus 2, 0, 2. The y-axis label is Parasite abundance, with tick labels 0, 200, 400, 600, 800. The legend title is Temp (degrees Celsius), with two entries labeled 18.2 and 20.2. One fitted curve slopes downward from left to right. The other fitted curve slopes upward from left to right. The two fitted curves intersect near Metals PC2 around 1 at a Parasite abundance around 200.

Navigate back to Figure 5.

## Table 1 Long description

The table summarizes seven fish species by average trophic level with standard error, average total length in millimeters with minimum and maximum, the number of individuals stored per jar, and the number dissected. Trophic level is lowest for Hybognathus nuchalis at about 2.0 and highest for Ictalurus punctatus at about 4.2, with other species between about 2.3 and 3.3. Mean total length is smallest for Gambusia affinis at about 33.7 mm and largest for Ictalurus punctatus at about 80.0 mm; the widest length range is Hybognathus nuchalis from 33 to 121 mm. Dissection counts are highest for Hybognathus nuchalis at 221 and lowest for Ictalurus punctatus at 88, with most others near 180 to 208. Jar loading varies from 2 individuals per jar for Carpiodes velifer and Percina vigil to 4 per jar for the remaining species. Comparisons should consider that trophic levels are reported as means with standard errors and may come from an external reference for Hybognathus species.

Navigate back to Table 1.

## Table 2 Long description

The table reports myxozoan parasite prevalence and mean abundance in multiple fish species sampled at a control site upstream and an impact site downstream of a pulp mill, with organs of infection listed for each parasite genus. The strongest signal is Myxobolus in Carpiodes velifer, with high prevalence at both sites (75.0 percent control, 67.0 percent impact) and similarly high mean abundance (85.37 versus 83.02 pseudocysts per fish). Henneguya in Ictalurus punctatus shows lower prevalence downstream (9.7 percent control, 5.3 percent impact) but much higher mean abundance downstream (0.13 versus 5.70). Several taxa show modest site differences: Myxobolus in Hybognathus nuchalis increases downstream (4.0 to 8.3 percent; mean abundance 0.08 to 0.38), while Unicauda in the same host decreases downstream (4.0 to 2.5 percent; mean abundance 0.58 to 0.17). Notropis atherinoides has similar Myxobolus prevalence at both sites (about 19 percent) but higher mean abundance downstream (1.44 to 3.63), and Pimephales vigilax shows lower Myxobolus prevalence downstream (14.8 to 9.5 percent) with slightly higher mean abundance (1.14 to 1.74). Chloromyxum and Myxidium are reported with prevalence only, and some mean abundance cells are blank, so comparisons for those entries are limited to prevalence.

Navigate back to Table 2.
