# Supplementary material for: Stressor interactions affect myxozoan abundance in a 42-year dataset from the Pearl River, Louisiana, USA
Source: Parasitology. 2026 Mar 25;153(5):654–66. doi: 10.1017/S0031182026101917 (PMC13315210; doi:10.1017/S0031182026101917)
Supplement: Díaz-Morales et al. supplementary material [file S0031182026101917sup001.docx]

**SUPPLEMENTARY MATERIAL**

Stressor interactions affect myxozoan abundance in a 42-year dataset from the Pearl River, Louisiana, USA

Dakeishla M. Díaz-Morales^1,2^, Stephen D. Atkinson^3^, Desmond Boyd^4^, Gabriella Commisso^2^, Shyanne Christner^5^, Imani Jones^6^, Katie Leslie^2^, Jolee Thirtyacre^2^, Connor J. Whalen^2^, Armand M. Kuris^7^, Justin Mann^8^, Henry Bart^9^, Chelsea L. Wood^2^

^1^DEPAUL UNIVERSITY, CHICAGO, IL, USA; ^2^ UNIVERSITY OF WASHINGTON, SEATTLE, WA, USA; ^3^ OREGON STATE UNIVERSITY, CORVALLIS, OR, USA; ^4^ UNIVERSITY OF SOUTH CAROLINA, COLUMBIA, SC, USA; ^5^ VALDOSTA STATE UNIVERSITY, VALDOSTA, GA, USA; ^6^ TUSKEGEE UNIVERSITY, AL, USA; ^7^ UNIVERSITY OF CALIFORNIA SANTA BARBARA, CA, USA; ^8^ TULANE UNIVERSITY BIODIVERSITY RESEARCH INSTITUTE, NEW ORLEANS, LA, USA; ^9^ TULANE UNIVERSITY, NEW ORLEANS, LA, USA

**Corresponding author:** Dakeishla M. Díaz-Morales, Email: ddiazmor@depaul.edu


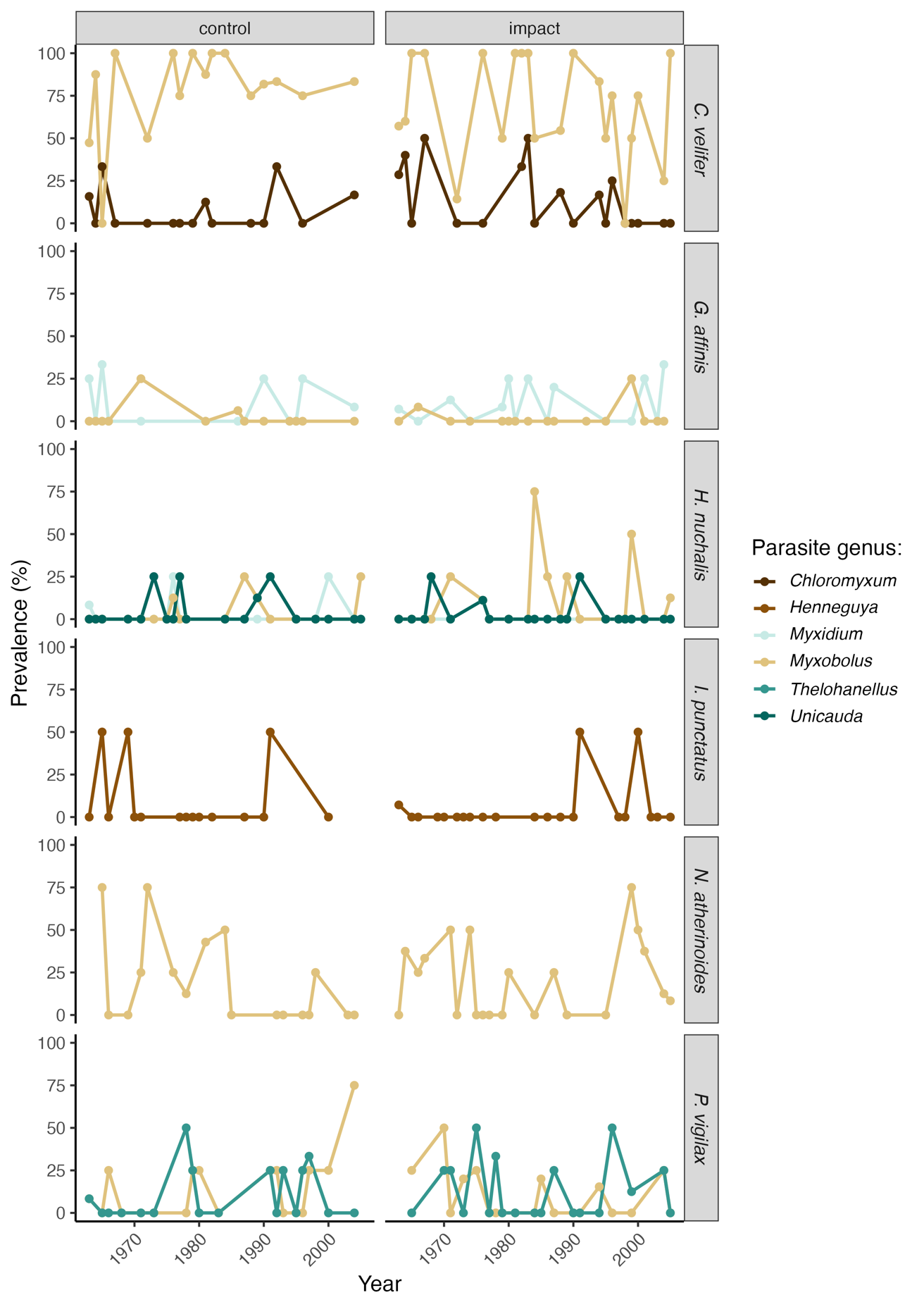


**Figure S1.** Change in prevalence of myxozoans across time (1963-2005) grouped by host species and site relative to a pulp mill outfall.


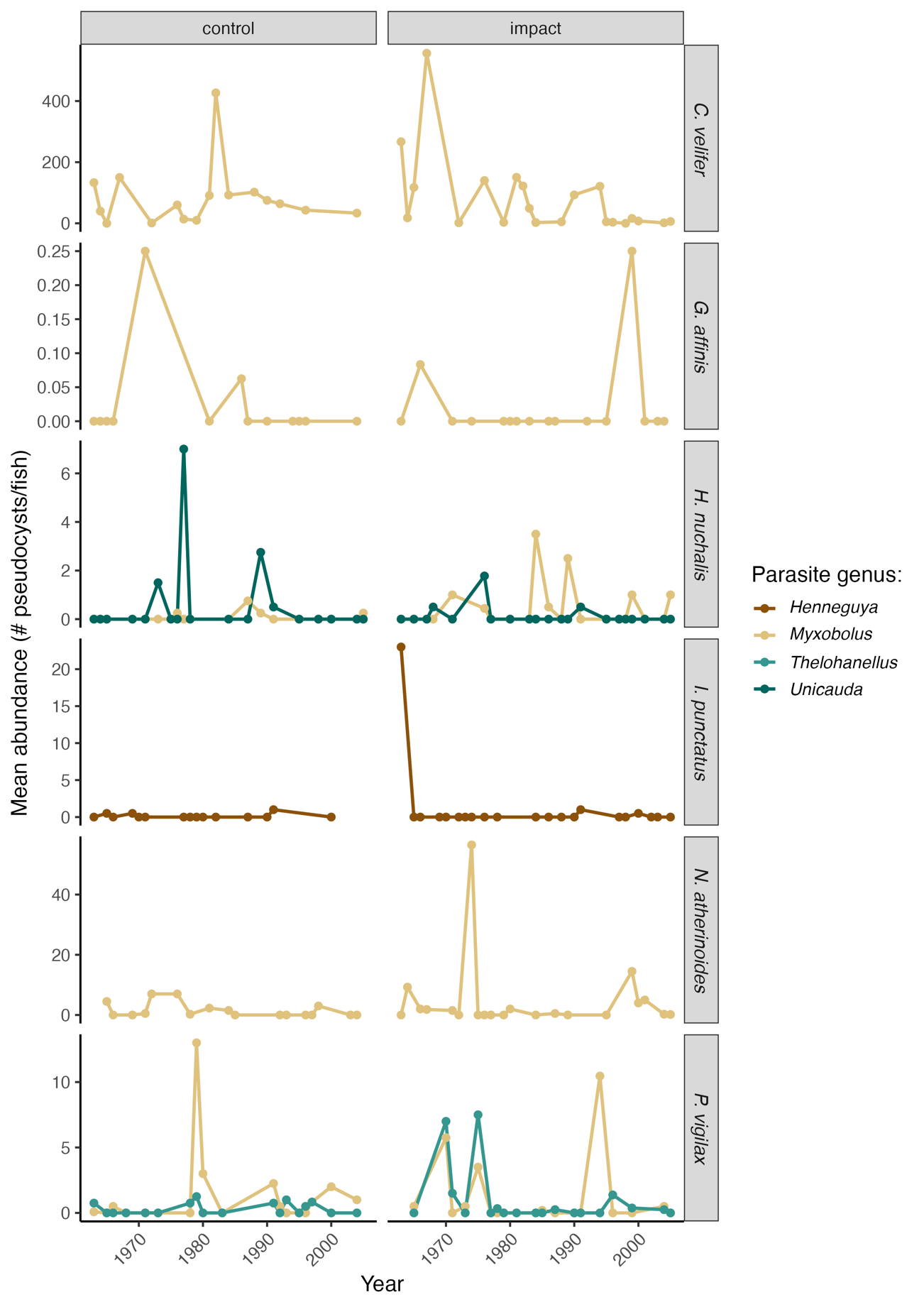


**Figure S2.** Change in mean abundance of myxozoans across time (1963-2005) grouped by host species and site relative to a pulp mill outfall.


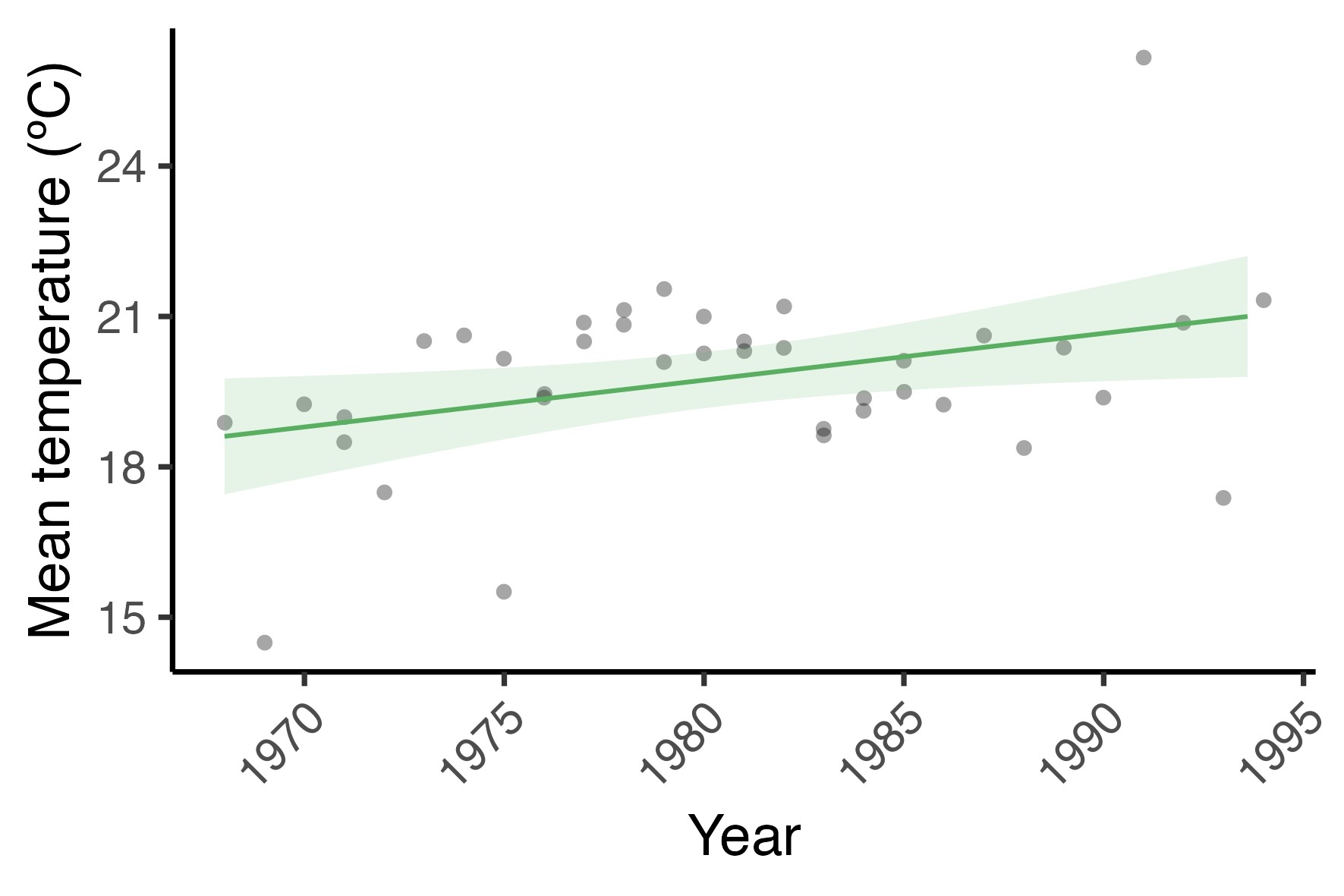


**Figure S3.** Mean temperature change based on linear model.


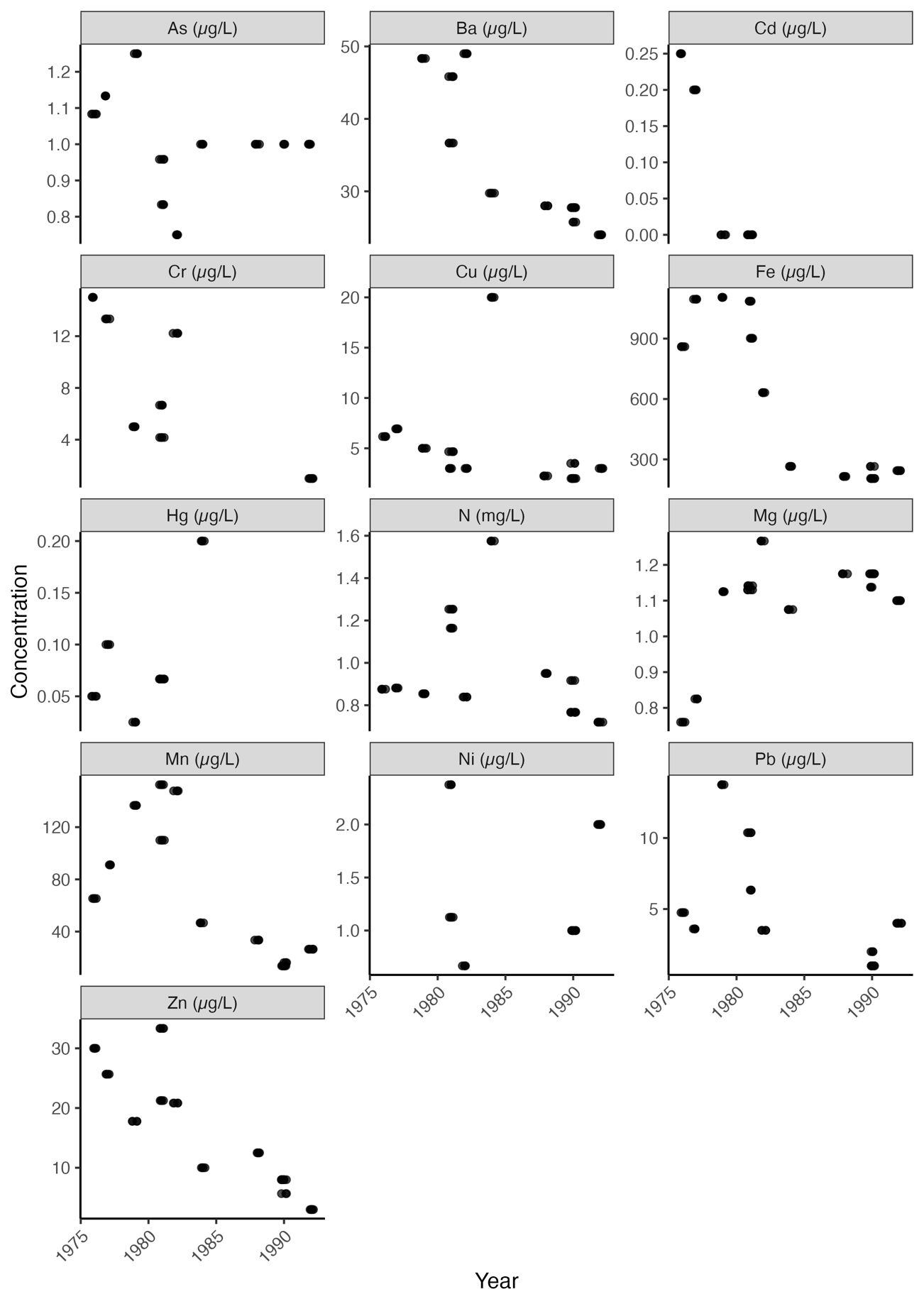


**Figure S4.** Changes in element and nitrogen concentration across time for the upstream gauge.

**Table S1:** Fish metadata summarized by the number of fish dissected at each site (including latitude and longitude) and with collection dates in human readable format (access here: https://github.com/wood-lab/TUBRI/blob/main/Manuscripts/Myxozoans/data/fish_metadata.csv).
